# Supplementary material for: Evaluating Translational Methods for Personalized Medicine—A Scoping Review
Source: J Pers Med. 2022 Jul 19;12(7):1177. doi: 10.3390/jpm12071177 (PMC9324577; doi:10.3390/jpm12071177)
Supplement: Supplementary file 1 [file jpm-12-01177-s001.zip › jpm-1763028-supplementary.pdf]

## Online Supplementary file 1 – Search strategy

## Pubmed

| **ONCOLOGY – 1/04/2020** | | |
|---|---|---|
| **No.** | **Query** | **Results** |
| #17 | #10 AND #6 AND #1 | 501 |
| #10 | #8 OR #9 | 57367 |
| #9 | Search: **Drug Evaluation, Preclinical [Majr]** "drug evaluation, preclinical"[MeSH Major Topic] | 20738 |
| #8 | Search: **"drug development"[tiab] OR "drug developments"[tiab] OR "drug response"[tiab] OR "Drug evaluation"[tiab] OR "Drug evaluated"[tiab] OR "drug response assay"[tiab] OR "drug sensitivity screening"[tiab])** | 37719 |
| #7 | Search: **Cancer*[tiab] OR carcinoma*[tiab] OR tumor*[tiab] OR tumour*[tiab] OR oncolo*[tiab] OR leukemia[tiab] OR lymphoma[tiab] OR sarcoma[tiab] OR "immune oncology" [tiab] OR "immunooncology" [tiab] OR Neoplasms [Majr])** | 4006907 |
| #6 | Search: **#4 OR #5** | 488511 |
| #5 | Search: **"Biomarkers"[Majr] OR "Precision Medicine"[Majr** "Biomarkers"[MeSH Major Topic] OR "Precision Medicine"[MeSH Major Topic] | 260230 |
| #4 | Search: **"stratified medicine"[tiab] OR biomarker*[tiab] OR "precision medicine"[tiab] OR "personalized medicine"[tiab] OR "personalised medicine"[tiab] OR "individualized medicine"[tiab] OR "individualised medicine"[tiab] OR "individualized therapy"[tiab] OR "individualised therapy"[tiab] OR "patient stratification"[tiab] OR "patient specific modeling"[tiab] OR "personalized clinical decision making"[tiab] OR "personalised clinical decision making"[tiab] OR "personalized clinical decision-making"[tiab] OR "prediction of response"[tiab]** | 279420 |
| #1 | Search: **"cellular model"[tiab] OR "cellular models"[tiab] OR "Patient specific modeling"[tiab] OR organoid*[tiab] OR "in silico"[tiab] OR "PDX models"[tiab] OR "Patient derived xenografts"[tiab] OR "preclinical PDX"[tiab] OR "humanised mouse model"[tiab] OR "preclinical models"[tiab] OR "preclinical model"[tiab] OR "pre clinical stage"[tiab] OR "pre clinical testing"[tiab] OR "Translational medical research"[tiab] OR "disease model"[tiab] OR "disease models"[tiab] OR "translational models"[tiab] OR "translational model"[tiab] OR "animal model"[tiab] OR xenograft*[tiab] OR "animal models"[tiab]** | |

| **BRAIN DISORDERS- 23/03/2020** | | |
|---|---|---|
| **No.** | **Query** | **Results** |
| #19 | Search **#7 AND #10 AND #13 AND #16** Sort by: **PublicationDate** Filters: **Publication date from 2005/01/01** | **678** |
| #18 | Search **#7 AND #10 AND #13 AND #16** | 714 |
| #16 | Search **#14 OR #15** | 1266325 |
| #15 | Search **("mental disorders" [Mesh] OR "Neurocognitive Disorders" [Mesh] OR "Neurodevelopmental Disorders" [Mesh] OR "Psychotic Disorders" [Mesh]))** | 1219103 |
| #14 | Search **("Psychiatric diseases" OR "mental disorders" OR "psychiatric disease" OR "psychiatric disorders" OR "psychiatric disorder" OR mental disorder" OR "mental illness" OR "depression OR "bipolar disorder" OR bipolar disorders" OR bipolarism OR anxiety OR "personality disorders" OR "psychotic disorders" OR schizophrenia OR "eating disorders" OR "trauma related disorders" OR "post traumatic stress disorder" OR "post traumatic stress disorders" OR "substance abuse disorders" OR "Asperger syndrome" OR Autism OR "Delirium tremens" OR Epilep* OR "Hallucinogen related disorders" OR Hysteria OR "Minor depressive disorder" "Minor depressive disorders" OR "Major depressive disorder" OR "Major depressive disorders" OR "Obsessive compulsive disorder" OR "Obsessive compulsive disorders" OR "Obsessive compulsive personality disorder" OR "Obsessive compulsive personality disorders" OR "Schizoaffective disorder" OR "Schizoaffective disorders" OR "Schizoid personality disorder"OR "Schizoid personality disorders" OR Alzheimer OR dementia)** | 219180 |
| #13 | Search **#11 OR #12** | 5947216 |

| | | |
|---|---|---|
| #12 | Search **drug therapy [Subheading]** | 2188351 |
| #11 | Search **(“therapy selection” “therapeutic selection” OR “treatment” OR “patient allocation” OR “drug therapy” OR “trial success rate” OR “therapy selected” OR “therapeutic selected”)** | 5947216 |
| #10 | Search **#8 OR #9** | 610949 |
| #9 | Search **(Search ("Biomarkers"[Mesh] OR "Precision Medicine"[Mesh]))** | 10235 |
| #8 | Search **((“stratified medicine” OR biomarker* OR “precision medicine” OR “personalized medicine” OR “personalised medicine” OR “individualized medicine“ OR “individualised medicine“ OR “individualized therapy“ OR “individualised therapy“ OR “patient stratification” OR OR “patient specific modeling” OR “personalized clinical decision making” OR “personalised clinical decision making”OR “personalized clinical decision-making” OR “prediction of response”))** | 607940 |
| #7 | Search **#5 OR #6** | 2760163 |
| #6 | Search **((Drug Evaluation, Preclinical [Mesh] OR Models, Animal [Mesh] OR Organoids [Mesh] OR In Vitro Techniques [Mesh] OR Translational Medical Research [Mesh] OR Disease models, animal [Mesh] OR Patient-specific modeling [Mesh] OR Cells, cultured [Mesh]))** | 2739423 |
| #5 | Search **((“cellular model” OR “cellular models” OR “drug development” OR “drug developments” OR ”drug response” OR “Drug evaluation” OR “Drug evaluated” OR “Patient specific modeling” OR organoid* OR ‘in silico’ OR ‘drug response assay” OR “drug sensitivity screening”OR “PDX models” OR “Patient derived xenografts” OR “preclinical PDX” OR “humanised mouse model” OR ”preclinical models” OR “preclinical model” OR “pre clinical stage” OR “pre clinical testing” OR “Translational medical research” OR “disease model” OR “disease models” OR “translational models”OR “translational model” OR “animal model” OR “animal models” OR xenograft*))** | 80945 |

## Embase

| **ONCOLOGY – 1/04/2020** | | |
|---|---|---|
| **No.** | **Query** | **Results** |
| #6 | #1 AND #2 AND #3 AND #4 AND [2005-2020]/py | **798** |
| #5 | #1 AND #2 AND #3 AND #4 | **801** |
| #4 | 'drug development':ti,ab OR 'drug developments':ti,ab OR 'drug response':ti,ab OR 'drug evaluation':ti,ab OR 'drug evaluated':ti,ab OR 'drug response assay':ti,ab OR 'drug sensitivity screening':ti,ab OR 'preclinical study'/exp/mj | **56857** |
| #3 | cancer*:ti,ab OR carcinoma*:ti,ab OR tumor*:ti,ab OR tumour*:ti,ab OR oncolo*:ti,ab OR leukemia:ti,ab OR lymphoma:ti,ab OR sarcoma:ti,ab OR 'immune oncology':ti,ab OR 'immunooncology':ti,ab OR 'neoplasm'/exp/mj | **5240665** |
| #2 | 'stratified medicine':ti,ab OR biomarker*:ti,ab OR 'precision medicine':ti,ab OR 'personalized medicine':ti,ab OR 'personalised medicine':ti,ab OR 'individualized medicine':ti,ab OR 'individualised medicine':ti,ab OR 'individualized therapy':ti,ab OR 'individualised therapy':ti,ab OR 'patient stratification':ti,ab OR 'patient specific modeling':ti,ab OR 'personalised clinical decision making':ti,ab OR 'personalized clinical decision making':ti,ab OR 'prediction of response':ti,ab OR 'biological marker'/exp/mj OR 'personalized medicine'/exp/mj | **435405** |
| #1 | 'cellular model':ti,ab OR 'cellular models':ti,ab OR 'patient specific modeling':ti,ab OR organoid*:ti,ab OR 'in silico':ti,ab OR 'pdx models':ti,ab OR 'patient derived xenografts':ti,ab OR 'preclinical pdx':ti,ab OR 'humanised mouse model':ti,ab OR 'preclinical models':ti,ab OR 'preclinical model':ti,ab OR 'pre clinical stage':ti,ab OR 'pre clinical testing':ti,ab OR 'translational medical research':ti,ab OR 'disease model':ti,ab OR 'disease models':ti,ab OR 'translational models':ti,ab OR 'translational model':ti,ab OR 'animal model':ti,ab OR xenograft*:ti,ab OR 'animal models':ti,ab | **427043** |
| **BRAIN DISORDERS – 24/03/2020** | | |
| #15 | #13 AND #14 | **680** |
| #14 | [embase]/lim NOT [medline]/lim | **9583919** |
| #13 | #3 AND #6 AND #9 AND #12 | **1133** |
| #12 | #10 OR #11 | **2126059** |

| | | |
|---|---|---|
| #11 | 'disorders of higher cerebral function'/exp/mj OR 'mental disease'/exp/mj OR 'psychosis'/exp/mj | **1448175** |
| #10 | 'psychiatric disease*':ti,ab OR 'mental disorder*':ti,ab OR 'psychiatric disorder*':ti,ab OR 'mental illness':ti,ab OR depression:ti,ab OR 'bipolar disorder':ti,ab OR bipolarism:ti,ab OR anxiety:ti,ab OR 'personality disorder*':ti,ab OR 'psychotic disorder*':ti,ab OR schizophreni*:ti,ab OR 'eating disorder*':ti,ab OR 'trauma related disorder*':ti,ab OR 'post traumatic stress disorder*':ti,ab OR 'substance abuse disorder*':ti,ab OR 'asperger syndrome':ti,ab OR autism:ti,ab OR 'delirium tremens':ti,ab OR epilep*:ti,ab OR 'hallucinogen related disorder*':ti,ab OR hysteria:ti,ab OR 'minor depressive disorder*':ti,ab OR 'major depressive disorder*':ti,ab OR 'obsessive compulsive disorder*':ti,ab OR 'obsessive compulsive personality disorder*':ti,ab OR 'schizoaffective disorder*':ti,ab OR 'schizoid personality disorder*':ti,ab OR alzheimer:ti,ab OR dementia:ti,ab | **1372445** |
| #9 | #7 OR #8 | **6083613** |
| #8 | 'drug therapy'/mj | **243267** |
| #7 | 'therapy selection':ti,ab OR 'therapeutic selection':ti,ab OR 'treatment':ti,ab OR 'patient allocation':ti,ab OR 'drug therapy':ti,ab OR 'trial success rate':ti,ab OR 'therapy selected':ti,ab OR 'therapeutic selected':ti,ab | **5925767** |
| #6 | #4 OR #5 | **433005** |
| #5 | 'biological marker'/exp/mj OR 'personalized medicine'/exp/mj | **93445** |
| #4 | 'stratified medicine':ti,ab OR biomarker*:ti,ab OR 'precision medicine':ti,ab OR 'personalized medicine':ti,ab OR 'personalised medicine':ti,ab OR 'individualized medicine':ti,ab OR 'individualised medicine':ti,ab OR 'individualized therapy':ti,ab OR 'individualised therapy':ti,ab OR 'patient stratification':ti,ab OR 'patient specific modeling':ti,ab OR 'personalized clinical decision making':ti,ab OR 'personalised clinical decision making':ti,ab OR 'prediction of response':ti,ab | **412990** |
| #3 | #1 OR #2 | **680024** |
| #2 | 'animal model'/exp/mj OR 'organoid'/exp/mj OR 'in vitro study'/exp/mj OR 'translational research'/exp/mj OR 'disease model'/exp/mj OR 'cell culture'/exp/mj OR 'preclinical study'/exp/mj | **236722** |
| #1 | 'cellular model*':ti,ab OR 'drug development*':ti,ab OR 'drug response':ti,ab OR 'drug evaluation':ti,ab OR 'drug evaluated':ti,ab OR 'patient specific modeling':ti,ab OR organoid*:ti,ab OR 'in silico':ti,ab OR 'drug response assay':ti,ab OR 'drug sensitivity screening':ti,ab OR 'pdx models':ti,ab OR 'patient derived xenografts':ti,ab OR 'preclinical pdx':ti,ab OR 'humanised mouse model':ti,ab OR 'preclinical model*':ti,ab OR 'pre clinical stage' OR 'pre clinical testing':ti,ab OR 'translational medical research':ti,ab OR 'disease model*':ti,ab OR 'translational model*':ti,ab OR 'animal model*':ti,ab OR xenograft*:ti,ab | **474356** |

## Web of Science

| **ONCOLOGY – 1/04/2020** | | |
|---|---|---|
| **No.** | **Query** | **Results** |
| #6 | #4 AND #3 AND #2 AND #1<br>**Refined by: PUBLICATION YEARS:** ( 2020 OR 2015 OR 2010 OR 2005 OR 2019 OR 2014 OR 2009 OR 2018 OR 2013 OR 2008 OR 2017 OR 2012 OR 2007 OR 2016 OR 2011 OR 2006 )<br>Indexes=SCI-EXPANDED, SSCI, A&HCI, CPCI-S, CPCI-SSH, ESCI Timespan=All years | 351 |
| #5 | #4 AND #3 AND #2 AND #1<br><br>Indexes=SCI-EXPANDED, SSCI, A&HCI, CPCI-S, CPCI-SSH, ESCI Timespan=All years | 358 |
| #4 | **TOPIC:** (“drug development” OR “drug developments” OR ”drug response” OR “Drug evaluation” OR “Drug evaluated” OR “drug response assay” OR “drug sensitivity screening”)<br><br>Indexes=SCI-EXPANDED, SSCI, A&HCI, CPCI-S, CPCI-SSH, ESCI Timespan=All years | 42100 |
| #3 | **TOPIC:** (Cancer* OR carcinoma* OR tumor* OR tumour* OR oncolo* OR leukemia OR lymphoma OR sarcoma OR “immune oncology” OR “immunooncology”)<br><br>Indexes=SCI-EXPANDED, SSCI, A&HCI, CPCI-S, CPCI-SSH, ESCI Timespan=All years | 3895924 |

| | | |
|---|---|---|
| #2 | **TOPIC:** ("stratified medicine" OR biomarker* OR "precision medicine" OR "personalized medicine" OR "personalised medicine" OR "individualized medicine" OR "individualised medicine" OR "individualized therapy" OR "individualised therapy" OR "patient stratification" OR "patient specific modeling" OR "personalized clinical decision making" OR "personalised clinical decision making" OR "personalized clinical decision making" OR "prediction of response")<br><br>Indexes=SCI-EXPANDED, SSCI, A&HCI, CPCI-S, CPCI-SSH, ESCI Timespan=All years | 357026 |
| #1 | **TOPIC:** ("cellular model" OR "cellular models" OR "Patient specific modeling" OR organoid* OR "in silico" OR "PDX models" OR "Patient derived xenografts" OR "preclinical PDX" OR "humanised mouse model" OR "preclinical models" OR "preclinical model" OR "pre clinical stage" OR "pre clinical testing" OR "Translational medical research" OR "disease model" OR "disease models" OR "translational models" OR "translational model" OR "animal model" OR xenograft* OR "animal models")<br><br>Indexes=SCI-EXPANDED, SSCI, A&HCI, CPCI-S, CPCI-SSH, ESCI Timespan=All years | 353567 |
| **BRAIN DISORDERS- 24/03/2020** | | |
| **No.** | **Query** | **Results** |
| # 12 | **#11 OR #6**<br><br>**Indexes=SCI-EXPANDED, SSCI, A&HCI, CPCI-S, CPCI-SSH, ESCI Timespan=All years** | 5 |
| # 11 | #10 AND #9 AND #8 AND #7<br><br>Indexes=SCI-EXPANDED, SSCI, A&HCI, CPCI-S, CPCI-SSH, ESCI Timespan=All years | 4 |
| # 10 | **TOPIC:** ('mental disease' OR 'disorders of higher cerebral function' OR 'mental disease' OR 'psychosis')<br><br>Indexes=SCI-EXPANDED, SSCI, A&HCI, CPCI-S, CPCI-SSH, ESCI Timespan=All years | 124,763 |
| # 9 | **TOPIC:** ('drug therapy')<br><br>Indexes=SCI-EXPANDED, SSCI, A&HCI, CPCI-S, CPCI-SSH, ESCI Timespan=All years | 373,565 |
| # 8 | **TOPIC:** ('biological marker' OR 'personalized medicine')<br><br>Indexes=SCI-EXPANDED, SSCI, A&HCI, CPCI-S, CPCI-SSH, ESCI Timespan=All years | 66,459 |
| # 7 | **TOPIC:** ('animal model*' OR organoid* OR 'in vitro study' OR 'translational research' OR 'disease model' OR 'cell culture' OR 'preclinical study'')<br><br>Indexes=SCI-EXPANDED, SSCI, A&HCI, CPCI-S, CPCI-SSH, ESCI Timespan=All years | 2,182,545 |
| # 6 | #5 AND #4 AND #3 AND #2 AND #1<br><br>Indexes=SCI-EXPANDED, SSCI, A&HCI, CPCI-S, CPCI-SSH, ESCI Timespan=All years | 1 |
| # 5 | **TITLE:** ("Psychiatric disease*" OR "mental disorder*" OR "psychiatric disorder*" OR "mental illness" OR depression OR "bipolar disorder" OR bipolarism OR anxiety OR "personality disorder*" OR "psychotic disorder*" OR schizophreni* OR "eating disorder*" OR "trauma related disorder*" OR "post traumatic stress disorder*" OR "substance abuse disorder*" OR "Asperger syndrome" OR Autism OR "Delirium tremens" OR Epilep* OR "Hallucinogen related disorder*" OR Hysteria OR "Minor depressive disorder*" OR "Major depressive disorder*" OR "Obsessive compulsive disorder*" OR "Obsessive compulsive personality disorder*" OR "Schizoaffective | 632,141 |

| | | |
|---|---|---|
| | disorder*" OR "Schizoid personality disorder*" OR Alzheimer OR dementia)<br><br>Indexes=SCI-EXPANDED, SSCI, A&HCI, CPCI-S, CPCI-SSH, ESCI Timespan=All years | |
| # 4 | **TITLE:** ("Psychiatric disease*" OR "mental disorder*" OR "psychiatric disorder*" OR "mental illness" OR depression OR "bipolar disorder" OR bipolarism OR anxiety OR "personality disorder*" OR "psychotic disorder*" OR schizophreni* OR "eating disorder*" OR "trauma related disorder*" OR "post traumatic stress disorder*" OR "substance abuse disorder*" OR "Asperger syndrome" OR Autism OR "Delirium tremens" OR Epilep* OR "Hallucinogen related disorder*" OR Hysteria OR "Minor depressive disorder*" OR "Major depressive disorder*" OR "Obsessive compulsive disorder*" OR "Obsessive compulsive personality disorder*" OR "Schizoaffective disorder*" OR "Schizoid personality disorder*" OR Alzheimer OR dementia)<br><br>Indexes=SCI-EXPANDED, SSCI, A&HCI, CPCI-S, CPCI-SSH, ESCI Timespan=All years | 632,141 |
| # 3 | **TITLE:** ("therapy selection" OR "therapeutic selection" OR "treatment" OR "patient allocation": ti,ab OR "drug therapy" OR "trial success rate" OR "therapy selected" OR "therapeutic selected")<br><br>Indexes=SCI-EXPANDED, SSCI, A&HCI, CPCI-S, CPCI-SSH, ESCI Timespan=All years | 1,122,829 |
| # 2 | **TITLE:** ("stratified medicine" OR biomarker* OR "precision medicine" OR "personalized medicine" OR "personalised medicine" OR "individualized medicine" OR "individualised medicine" OR "individualized therapy" OR "individualised therapy" OR "patient stratification" OR "patient specific modeling" OR "personalized clinical decision making" OR "personalised clinical decision making" OR "prediction of response")<br><br>Indexes=SCI-EXPANDED, SSCI, A&HCI, CPCI-S, CPCI-SSH, ESCI Timespan=All years | 107,628 |
| # 1 | **TITLE:** ("cellular model*" OR "drug development*" OR "drug response" OR "Drug evaluation" OR "Drug evaluated" OR "Patient specific modeling" OR organoid* OR "in silico" OR "drug response assay" OR "drug sensitivity screening" OR "PDX models" OR "Patient derived xenografts" OR "preclinical PDX" OR "humanised mouse model" OR "preclinical model*" OR "pre clinical stage" OR "pre clinical testing" OR "Translational medical research" OR "disease model*" OR "translational model*" OR "animal model*" OR xenograft*)<br><br>Indexes=SCI-EXPANDED, SSCI, A&HCI, CPCI-S, CPCI-SSH, ESCI Timespan=All years | 78,144 |

| Date of search | Source | Url |
|---|---|---|
| 11/05/2020 | EUROPEAN ALLIANCE FOR PERSONALIZED MEDICINE | https://www.euapm.eu |
| 01/2021 | International register of preclinical trial protocols | www.preclinicaltrials.eu |
| 01/2021 | Animal Study Registry | www.animalstudyregistry.org |
| 01/2021 | Safe, Innovative and Accessible Medicines: a Renewed Vision for the Pharmaceutical Sector | https://eur-lex.europa.eu/LexUriServ/LexUriServ.do?uri=COM:2008:0666:FIN:en:PDF |
| 01/2021 | Gyoerffi M. ENVIRONMENT, PUBLIC HEALTH AND FOOD | https://www.europarl.europa.eu/RegData/etudes/BRIE/2017/614190/IPOL_BRI(2017)614190_EN.pdf |

| | | |
|---|---|---|
| | SAFETY (ENVI) Briefing Personalised Medicine-Current Status KEY FINDINGS. | |

## Grey Literature

**Online Supplementary file 2 – Data extraction form**

## ONCOLOGY:

<table>
<tr><td>Authors</td><td colspan="2"></td></tr>
<tr><td>Title</td><td colspan="2"></td></tr>
<tr><td>Journal</td><td colspan="2">Year of Publication</td></tr>
<tr><td>Country of origin</td><td colspan="2"></td></tr>
<tr><td>Date of publication</td><td colspan="2"></td></tr>
<tr><td>DOI</td><td colspan="2"></td></tr>
<tr><td>Language</td><td colspan="2">• English<br>• French<br>• German<br>• Spanish<br>• Italian</td></tr>
<tr><td>Type of publication</td><td colspan="2">• Research article<br>• Systematic review<br>• Review<br>• Commentary/ Editorial<br>• Book chapter</td></tr>
<tr><td>Disease</td><td colspan="2">• Type of cancer<br>• Other than oncology</td></tr>
<tr><td>Topic</td><td colspan="2">Short description</td></tr>
<tr><td rowspan="4">Type of model</td><td>Animal</td><td>• Animal species<br>• Type of animal model<br>• Origin of tumour<br>• Immune status</td></tr>
<tr><td>Cellular model</td><td>• Type of cellular model<br>• Origin of cells</td></tr>
<tr><td>Organoid model</td><td>Origin of organoid</td></tr>
<tr><td>In silico models</td><td>• Type of in silico model<br>• Type and source of data</td></tr>
<tr><td>Advantages</td><td colspan="2">Advantages of the preclinical model</td></tr>
<tr><td>Disadvantages</td><td colspan="2">Disadvantages of the preclinical model</td></tr>
<tr><td>Validity</td><td colspan="2">Has the model been validated (external and internal validation)</td></tr>
<tr><td>Personalised medicine</td><td colspan="2">Consideration related to personalised medicine</td></tr>
</table>

**BRAIN DISORDERS:**

| | |
|---|---|
| **Title** | |
| **Year** | Year of Publication |
| **Authors** | |
| **Journal** | |
| **DOI** | |
| **Language** | • English<br>• French<br>• German<br>• Spanish<br>• Italian |
| **Type of paper** | • Research article<br>• Review<br>• Congress report / abstract<br>• Commentary/ Editorial<br>• Book chapter |
| **Disease** | • Psychiatric disorders<br>• Trauma-related disorders<br>• Substance use disorders<br>• Anxiety disorders<br>• Neurodegenerative disorders (Alzheimer's disease, Parkinson's disease, Huntington's disease)<br>• Neurodevelopmental disorders (Autism spectrum diseases)<br>• Others |
| **Type of model** | • Animal – rodent<br>• Animal – others<br>• Cellular model<br>• Organoids<br>• *In silico* models |
| **Pro** | • Advantages of the preclinical model |
| **Cons** | • Disadvantages of the preclinical model |
| **Face validity** | Does the model resemble the human disease condition on a superficial level, for example, biochemistry or symptomatology? |
| **Predictive validity** | Can the model successfully discriminate between successful and unsuccessful treatments for the human disease condition? |
| **Personalised medicine** | Consideration related to personalised medicine |
